# The Pif1 Helicase, a Negative Regulator of Telomerase, Acts Preferentially at Long Telomeres

**DOI:** 10.1371/journal.pgen.1005186

**Published:** 2015-04-23

**Authors:** Jane A. Phillips, Angela Chan, Katrin Paeschke, Virginia A. Zakian

**Affiliations:** 1 Princeton University, Department of Molecular Biology, Princeton, New Jersey, United States of America; 2 University of Würzburg, Department of Biochemistry, Biocenter, Würzburg, Germany; Columbia University Medical Center, UNITED STATES

## Abstract

Telomerase, the enzyme that maintains telomeres, preferentially lengthens short telomeres. The *S*. *cerevisiae* Pif1 DNA helicase inhibits both telomerase-mediated telomere lengthening and *de novo* telomere addition at double strand breaks (DSB). Here, we report that the association of the telomerase subunits Est2 and Est1 at a DSB was increased in the absence of Pif1, as it is at telomeres, suggesting that Pif1 suppresses *de novo* telomere addition by removing telomerase from the break. To determine how the absence of Pif1 results in telomere lengthening, we used the single telomere extension assay (STEX), which monitors lengthening of individual telomeres in a single cell cycle. In the absence of Pif1, telomerase added significantly more telomeric DNA, an average of 72 nucleotides per telomere compared to the 45 nucleotides in wild type cells, and the fraction of telomeres lengthened increased almost four-fold. Using an inducible short telomere assay, Est2 and Est1 no longer bound preferentially to a short telomere in *pif1* mutant cells while binding of Yku80, a telomere structural protein, was unaffected by the status of the *PIF1* locus. Two experiments demonstrate that Pif1 binding is affected by telomere length: Pif1 (but not Yku80) -associated telomeres were 70 bps longer than bulk telomeres, and in the inducible short telomere assay, Pif1 bound better to wild type length telomeres than to short telomeres. Thus, preferential lengthening of short yeast telomeres is achieved in part by targeting the negative regulator Pif1 to long telomeres.

## Author Summary

Telomerase, the enzyme that maintains telomeres, preferentially lengthens short telomeres. The baker’s yeast Pif1 DNA helicase inhibits both telomerase-mediated lengthening of existing telomeres and the formation of new telomeres at double strand breaks. By virtue of its ATPase activity, Pif1 reduces the level of telomerase binding to telomeres. Here, we report that the association of the telomerase subunits Est2 and Est1 at a DNA break was increased in the absence of Pif1, suggesting that Pif1 affects telomere length and new telomere formation by similar mechanisms. In cells lacking Pif1, Est2 and Est1 no longer bound preferentially to short telomeres, a larger fraction of telomeres was lengthened and the amount of telomeric DNA added per telomere was increased compared to wild type cells. Furthermore, by two different assays, Pif1 bound preferentially to long telomeres *in vivo*. Thus, preferential lengthening of short telomeres is achieved in part by targeting Pif1, a negative regulator of telomerase, to long telomeres.

## Introduction

Telomerase is a specialized reverse transcriptase that extends the G-strand of telomeric DNA using its RNA subunit as a template. In *Saccharomyces cerevisiae*, telomerase consists minimally of Est2, the catalytic reverse transcriptase, TLC1, the templating telomerase RNA, and Est1 and Est3, two telomerase accessory subunits that are both essential for telomerase action *in vivo*. In addition, yeast telomerase requires Cdc13, the sequence specific TG_1-3_-binding subunit of the CST (Cdc13-Stn1-Ten1) complex that has dual roles in protecting telomeres from degradation and recruiting telomerase to DNA ends (reviewed in [1]).

Yeast telomerase is regulated by both the cell cycle and telomere length. Telomerase-mediated telomere lengthening occurs only in late S/G2 phase, even though telomerase activity is present throughout the cell cycle [2, 3]. Although Est2 and TLC1 are telomere associated even in G1 phase when telomerase is not active, Est1 [4] and Est3 [5] come to the telomere primarily in late S/G2 phase. Short telomeres are preferentially lengthened by telomerase [6, 7], a pattern that can be explained by higher levels of telomerase binding to short telomeres late in the cell cycle [8, 9]. Both the Tel1 checkpoint kinase [8] and Tbf1 [10], a telomere structural protein that binds to the sub-telomeric DNA of some telomeres, target telomerase to short telomeres. Tel1 itself binds preferentially to short telomeres [8, 11], as does the Mre11-Rad50-Xrs2 (MRX) complex [12], which recruits Tel1 to telomeres [8]. Short telomeres have reduced levels of Rif2 [8, 12], a telomere structural protein that negatively regulates telomerase [13, 14]. Because Rif2 and the MRX complex compete with each other for telomeric DNA binding [15], in the absence of Rif2, Tel1 no longer binds preferentially to short telomeres [12].

The *S*. *cerevisiae* Pif1 is the founding member of a helicase family that exists in virtually all eukaryotes (reviewed in [16]). Pif1 was first identified because of its important role in maintaining mitochondrial DNA [17]. However, there are two forms of Pif1 depending on whether the first or second AUG is used to start translation, one destined for mitochondria and one localized to nuclei [18, 19]. Nuclear Pif1 inhibits telomerase at both telomeres and double strand breaks (DSBs) [18–20]. Thus, telomeres are longer and the rate of *de novo* telomere addition to DSBs is greatly elevated in *pif1* mutant cells. Checkpoint-mediated phosphorylation of Pif1 is required for Pif1 inhibition of telomerase at DSBs but not at telomeres [21]. *In vivo* and *in vitro*, Pif1 uses its ATPase activity to displace telomerase from DNA ends [22]. Pif1 also has more general roles in chromosome maintenance: it facilitates replication and suppresses DNA damage at G-quadruplex motifs, cooperates with Dna2 to process long Okazaki fragments, is needed for stability of mitochondrial DNA, and promotes break-induced replication (BIR) (reviewed in [16]; also, [23–25]).

Here, we show that Pif1 acts similarly at DSBs and telomeres in that its presence was associated with lower levels of telomerase binding at DSBs (as it is at telomeres [22]). Using an assay that monitors telomeric DNA addition at individual telomeres [7], we find that Pif1 reduced both the frequency and processivity of telomere addition. Moreover, telomerase was no longer bound preferentially to short telomeres in Pif1-deficient cells. By two assays, Pif1 bound preferentially to long telomeres. Together, these data can be explained if Pif1 is more likely to remove telomerase from those telomeres that are least in need of lengthening.

## Results

### Pif1 reduces telomerase association at DSBs

**Fig 1:**
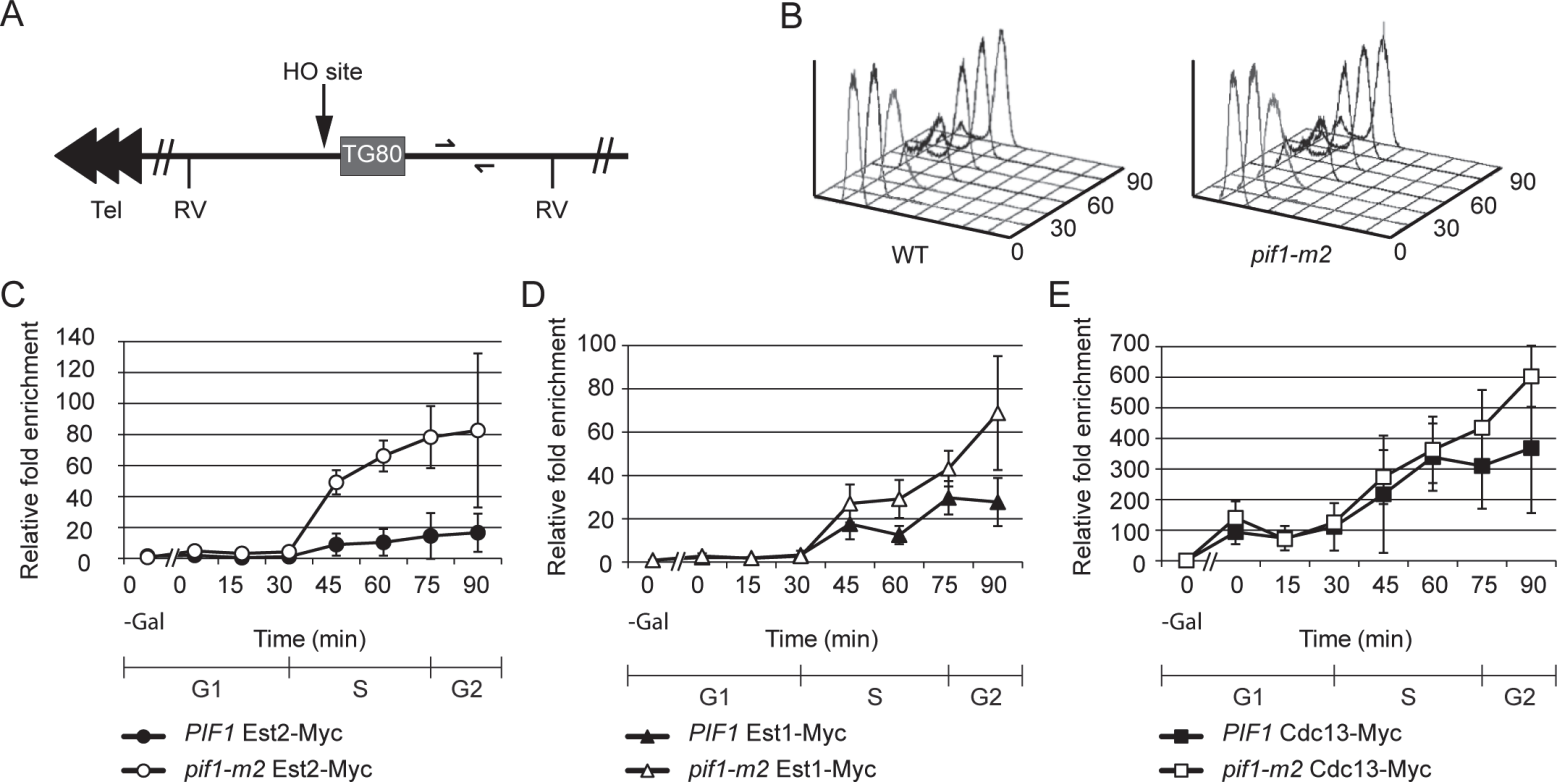
Telomerase, but not Cdc13, association is increased at a double strand break in *pif1-m2* cells. (A) Chromosome VII-L was modified by inserting an *HO* endonuclease recognition site and an adjacent 80 bp tract of TG_1-3_ DNA ~13 kb from the left telomere [26]. Half arrows indicate unique sequences used for real-time PCR amplification. Each arrowhead represents ~100 bps C_1-3A_/TG_1-3_ DNA. RV, *Eco*RV recognition site. (B) Cell cycle progression was monitored with flow cytometry. (C) Results were quantitated using real-time PCR. Est2 binding was significantly increased in *pif1-m2* (open circles) compared to WT cells (closed circles) at 45, 60, and 75 min (p = 0.0018 to 0.011; two-tailed unpaired *t*-tests). (D) A modest but reproducible increase in Est1 binding in *pif1-m2* cells during and after S phase was observed in four independent synchronies: at 60, 75, and 90 min, Est1 binding in *pif1-m2* (open triangles) was 1.1–3.6x (average 2.3x), 1.2–2.3x (average 1.5x), and 2.1–3.4x (average 2.5x) increased over that in WT cells (closed triangles), respectively. This increase was significant at 60 and 90 min (p = 0.014, 0.028). Overlapping standard deviations at the 75 min time point (p = 0.054) and all large standard deviations are due to slight differences in *HO* cutting efficiency in four independent experiments. (E) Cdc13 binding in WT (closed squares; as shown previously in [12]) and *pif1-m2* (open squares) cells was statistically indistinguishable.

Pif1 uses its ATPase activity to evict telomerase from telomeres [22], which can explain how it suppresses telomere lengthening. Pif1 also inhibits telomere addition to DSBs [19, 20]. To determine if Pif1 affects telomerase binding to DSBs, we used chromatin immuno-precipitation (ChIP) to monitor the association of Est1 and Est2 to an induced DSB in the presence and absence of Pif1 (Fig 1). These experiments were carried out in a strain with a galactose-inducible HO endonuclease and an *HO* recognition site ~13 kb from the end of chromosome VII-L, the only accessible *HO* site in the strain (Fig 1A) [26]. We used a strain with an 80-bp tract of TG_1-3_ telomeric DNA (TG80, Fig 1A grey box) adjacent to the *HO* site to increase the rate of *de novo* telomere addition [26]. Cells also expressed a Myc-tagged protein (Est2, Est1, or Cdc13). Experiments were carried out in *PIF1* and *pif1-m2* versions of the strain, where *pif1-m2* cells express wild type (WT) levels of mitochondrial Pif1 and reduced nuclear Pif1 [18]. Although *pif1-m2* retains some nuclear function, we used it because *pif1-m2* cells progress through the cell cycle similarly to WT cells, unlike *pif1Δ* cells which progress more slowly owing to their reduced mitochondrial function [19]. In addition, *pif1Δ* cells are very hard to synchronize. The efficiency of cleavage at the *HO* site, which was monitored by Southern blotting, was not affected by Pif1 levels (~65–80% cutting in both WT and *pif1-m2* cells; S1 Fig).

HO cleavage was induced in WT or *pif1-m2* cells that were first arrested in late G1 phase with Δ-factor. After HO action, cells were released from the G1 phase arrest. In both strains, ChIP samples were taken in G1 arrested cells both before (“0—gal” time points) and after HO induction and throughout the synchronous cell cycle that occurred upon removal from Δ-factor (0–90 min time points; 24°C).

Samples from each time point were also assessed by FACS to determine cell cycle position, which demonstrated that *pif1-m2* and WT cells moved similarly through the cell cycle (Fig 1B). ChIP samples were quantified using real-time PCR and normalized to input DNA. In this and other ChIP experiments, results are presented as fold enrichment of binding to the DSB compared to binding to a control site (*ARO1*). We examined binding of Est2 (Fig 1C), Est1 (Fig 1D) and Cdc13 (Fig 1E) to the *HO* break site in WT and *pif1-m2* cells.

None of the three proteins was associated with the *HO* recognition site before HO induction (Fig 1C–1E, 0—gal time point). In both strains, Est2 binding to TG80-HO was at background levels in G1 and early S phase (Fig 1C, WT closed squares; *pif1-m2*, open squares, 0–30 min time points). In both strains, high levels of Est2 binding to the DSB were detected from mid-S phase through the end of the cell cycle (45–90 min time points). However, average Est2 binding was over four times higher in *pif1-m2* compared to WT cells (Fig 1C, open circles). Est1 showed a similar pattern of binding to the DSB, occurring at background levels in G1 and early S phase with strong binding from mid-S phase to the end of the cell cycle (Fig 1D). Although Est1 binding was significantly higher in *pif1-m2* versus WT cells from mid-S to the end of the cell cycle, the difference was fairly modest (~1.5–2.5-fold over WT levels; see Fig 1 legend for p values). As reported previously [12], Cdc13 showed strong association with TG80-HO after HO induction. Cdc13 enrichment at TG80-HO was ~100 to 150-fold over background during G1 phase and steadily increased to ~400- to 600-fold over background by the end of the cell cycle. However, unlike Est2 and Est1, Cdc13 binding to TG80-HO was not affected significantly by reduced Pif1 (Fig 1E, open squares).

We conclude that Pif1 affects telomere addition to DSBs by reducing telomerase binding to breaks, as it does at telomeres. This effect is specific for telomerase, as binding of Cdc13 was unaffected by Pif1 at either telomeres [22] or DSBs (Fig 1E).

### Pif1 reduces the fraction of elongated telomeres and amount of telomerase-mediated telomere elongation

**Fig 2:**
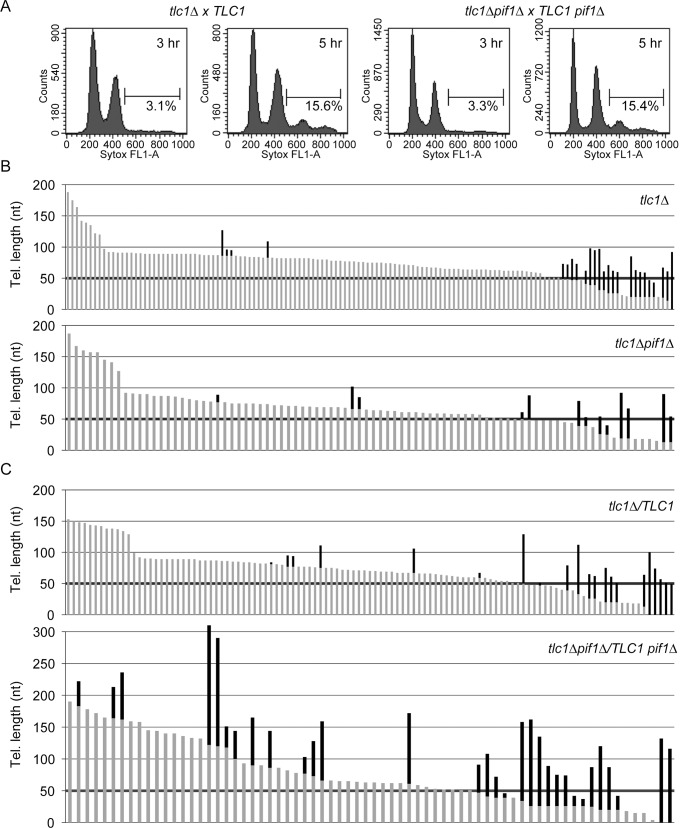
Absence of Pif1 results in increased frequency and extent of telomerase lengthening. (A) Flow cytometry was used to measure DNA content of *PIF1 tlc1Δ* and *pif1Δ tlc1Δ* recipient samples (after mating with *PIF1 TLC1* donor or *pif1Δ TLC1* donor cells, respectively) at 3 and 5 h post-mixing. Percentages indicate cells with a 4C DNA content, which corresponds to cells that have gone through one S phase after mating. ~93% of the recipient cells mated in both crosses, and replication of the diploid genomes occurred by 5 h post-mixing (15.5% out of an expected 16.7% of cells had 4C DNA content at this time). Thus, the null status of *PIF1* did not affect growth rates or mating efficiency during the small number of generations required to obtain sufficient cells for this experiment. (B) Telomere length is plotted; individual telomeres are shown as vertical lines (gray, parental telomeric sequence; black, telomere lengthening events). Telomere VII-L was sequenced from telomerase-deficient *tlc1Δ* (top; n = 134 telomeres) and *tlc1Δ pif1Δ* (bottom; n = 86 telomeres) haploid cells after approximately 30 doublings following dissection from JP192 and JP223, respectively. Telomere elongation frequency (p = 0.712; Fisher’s exact test) and length of telomeric sequence added (p = 0.543; Mann-Whitney U test) are indistinguishable between the two strains. (C) Telomere VII-L was sequenced after the first post-zygotic S phase from *tlc1Δ /TLC1* (top; n = 111 telomeres) and *tlc1Δ pif1Δ /TLC1 pif1Δ* (bottom; n = 70 telomeres) diploid cells 5 h after reintroduction of telomerase by mating to telomerase-deficient strains. The frequency of telomere elongation in *tlc1Δ pif1Δ /TLC1 pif1Δ* cells is significantly increased over *tlc1Δ /TLC1* cells by ~2-fold (p = 0.000180; Fisher’s exact test). Additionally, the average length of added telomeric sequence is significantly increased (p = 0.0265; Mann-Whitney U test) in *tlc1Δ pif1Δ/TLC1 pif1Δ* cells (72 nt) compared with *tlc1Δ/TLC1* cells (43 nt). For each cross, telomere sequencing data are compiled results from two independent experiments. Due to high amount of telomerase independent elongation events at very short telomeres, we excluded telomeres of 50 bps or shorter in determining both the frequency and extent of elongation (threshold is indicated by bold horizontal lines).

Pif1-mediated removal of telomerase could regulate telomere length by affecting the frequency of telomere addition or telomerase processivity (or both). To distinguish among these possibilities, we used the Single Telomere Extension (STEX) assay [7], which analyzes lengthening at individual telomeres at nucleotide resolution over a single cell cycle in diploid *PIF1* and *pif1Δ* cells (Fig 2). Freshly dissected *pif1Δ* spore clones were used quickly after dissection from a heterozygous diploid to minimize the negative effects of mitochondrial DNA loss on growth rate.

Telomerase-deficient *tlc1Δ PIF1* or *tlc1Δ pif1Δ* spore clones (recipient cells) were mated to telomerase proficient *TLC1 PIF1* or *TLC1 pif1Δ* cells (donor cells), respectively, to restore telomerase activity in the resulting diploids. To distinguish recipient from donor telomeres, *URA3* was integrated adjacent to the left telomere of chromosome VII in the recipient strain. Mating efficiencies and cell cycle progression, which were determined by flow cytometry (Fig 2A), were similar in the two crosses (Fig 2A). ~93% of the recipient cells mated in both crosses (Fig 2A) and replication of the diploid genomes occurred by 5 h post-mixing (15.5% out of an expected 16.7% of cells had 4C DNA content at this time; see Fig 2A legend). Thus, the null status of *PIF1* did not affect growth rates or mating efficiency during the small number of generations required to obtain sufficient cells for this experiment. The *URA3*-marked VII-L telomeres derived from recipient cells were amplified, cloned, and sequenced. Because yeast telomerase adds irregular TG_1-3_ repeats [27], pre-existing telomeric DNA can be distinguished from the newly added sequence [7, 28].

Prior to mating, we measured telomere lengthening in the parental haploid *tlc1Δ PIF1* (Fig 2B, top) and *tlc1Δ pif1Δ* (Fig 2B, bottom) spore clones to obtain an estimate of the frequency of lengthening in the absence of telomerase, which likely occurs *via* recombination. In both strains, many of the short telomeres (≤50 bps) were lengthened in the absence of telomerase. Among longer telomeres (≥50 bps), the fraction of telomeres lengthened (3.7% and 4.8%) by telomerase-independent events and the average amount of telomeric DNA added (39 and 35 nt) were similar in, respectively, *tlc1Δ* and *tlc1Δ pif1Δ* haploid cells. To limit analysis to telomeres lengthened by telomerase, we considered only telomeres longer than 50 bps for further analysis.

Next, we prepared DNA from the diploid cells at the end of the first post-zygotic S phase in both WT (Fig 2C, top) and *pif1Δ* (Fig 2C, bottom) cells. The elongation frequency for telomere VII-L in *tlc1Δ pif1Δ/TLC1 pif1Δ* was 28% for telomeres over 50 bp. In contrast, only 7.2% of the telomeres were lengthened in WT cells, a significant difference (see Fig 2 legend for p values). Moreover, the average length of telomeric sequence added to individual telomeres in *tlc1Δ pif1Δ/TLC1 pif1Δ* cells was 72 nt, a value significantly greater than 45 nt that was added in *PIF1* cells (see Fig 2 legend for p values). The value obtained in *PIF1* WT (*tlc1Δ/TLC1*) cells in our experiments was the same as reported previously for STEX in *tlc1Δ/TLC1* cells [7]. Taken together, these data suggest that Pif1 reduces both the frequency and processivity of telomerase action.

### Pif1 is required for preferential binding of telomerase to short telomeres

**Fig 3:**
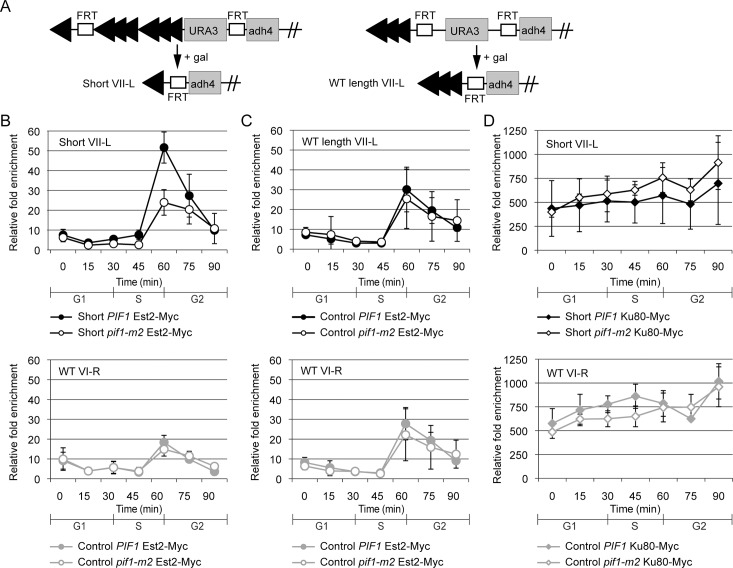
Preferential S-phase Est2 binding at short telomeres is decreased in *pif1-m2* cells. (A) Schematic of modified chromosome VII-L inducible short telomere constructs. In these strains, Flp1 causes recombination between the two FRT sites. Each arrowhead represents ~100 bp C_1-3A_/TG_1-3_ DNA. The 34 bp FRT recognition sites are labeled. Flp1 action generates a ~90 bp telomere (“Short VII-L”) in a WT strain [8]. A control strain (“WT length VII-L”) in which Flp1 action generates a ~300 bp telomere was also used [6]. (B) The lengths and identities of the telomeres being studied are indicated in the upper left corner of each graph. Binding of Est2-Myc at short telomere VII-L (top) was significantly decreased in *pif1-m2* (open circles) compared with WT cells (closed circles) during S phase (45 min, p = 0.025; 60 min, p = 0.0092; two-tailed unpaired *t*-tests). Binding of Est2 at WT length telomere VI-R (bottom) in the same cells was statistically indistinguishable in *pif1-m2* and WT cells. Although Est2 binds telomeres in G1 and early S phase, this binding is not evident in these experiments because it occurs mainly in the subtelomeric region that is deleted by FLP action, rather than at the very end of the chromosome [8]. (C) Binding of Est2 at WT length telomere VII-L (top) was statistically indistinguishable between *pif1-m2* (open circles) and WT (closed circles) cells. Binding of Est2 at WT length telomere VI-R (bottom) in the same cells was unaffected by the *pif1-m2* mutation. (D) Binding of Ku80-Myc at short telomere VII-L (top) was statistically indistinguishable in *pif1-m2* (open diamonds) compared with WT cells (closed diamonds). Ku80 binding at WT length telomere VI-R (bottom) in the same cells was statistically indistinguishable in *pif1-m2* and WT cells.

In earlier work, we did not see a difference in Est2 binding to bulk telomeres in asynchronous cells [12]. To explore the effects of Pif1 on telomerase binding, we combined an inducible short telomere system [6] with ChIP in cells going synchronously through S phase (see [8] and Fig 3). The experimental strains used in these experiments had a modified chromosome VII-L in which a cassette containing telomeric repeats flanked by recognition sites (FRT) for the Flp1 site-specific recombinase was positioned directly adjacent to the telomere (Fig 3A left, experimental strain), as well as an integrated copy of a galactose-inducible *FLP1*. The control strains were isogenic to the experimental strains except that the cassette at modified chromosome VII-L had no telomeric DNA between the two FRT sites [6] (Fig 3A, right, control strain). In both strains, *FLP1* expression was induced by adding galactose, which caused recombination between the two FRT sites. In the experimental strain, FLP action resulted in loss of the telomeric DNA between the sites, thus generating a short ~100 bp telomere at chromosome VII-L that is preferentially lengthened by telomerase for multiple cell cycles [6]. The telomere adjacent to the control cassette is maintained at the strain characteristic length (~300 bp in WT cells) regardless of whether or not it is acted upon by Flp1 (Fig 3A). Both the experimental and control strains expressed a Myc-tagged protein (Est1, Est2 or Yku80). These experiments were carried out in parallel in WT and *pif1-m2* versions of both the experimental and control strains. Cells were synchronized, and ChIP samples were prepared and quantified as described for the DSB experiments.

Galactose was added to G1-arrested cells to induce expression of Flp1, which caused recombination at the VII-L telomeric region in all strains, resulting in either a short VII-L telomere (experimental strain) or a VII-L telomere that was the same length as the other telomeres in the cell (control strain). The efficiency of recombination was similar in all strains (S2A Fig). After Flp1 action, cells were released from G1 arrest (time 0), and samples were removed for analysis throughout the first synchronous cell cycle (15 to 90 min). The efficiency of recombination, which was monitored in each experiment (S2A Fig), was equivalent (~75%) in WT and *pif1-m2* cells for both the experimental and control strains.

Using the same system, previous studies found that Est2 and Est1 binding was approximately two times higher on the short VII-L telomere versus the WT length VI-R telomere in the same cells or the WT length VII-L or VI-R telomeres in the control strain [8, 9]. For this study, we recapitulate the preferential binding of Est2 and Est1 to the short VII-L telomere in WT cells (Figs 3B and S3). Notably, preferential Est2 binding to the shortened VII-L telomere was lost in *pif1-m2* cells (Fig 3B, upper panel, open circles), even though the amount of Est2 binding to the unaltered VI-R telomere in the same cells was unchanged (Fig 3B lower panel, see legend for p values). In addition, levels of Est2 binding were the same in *pif1-m2* and WT versions of the control strain at the VII-L telomere (Fig 3C, upper panel). Similar results were obtained for Est1 (S3 Fig).

To determine if Pif1 acts preferentially on telomerase binding or alternatively affects all telomere-binding proteins, we examined Myc-tagged Ku80 telomere association as a function of Pif1 (Fig 3D). Consistent with earlier results [8], Yku80 bound robustly and equally well to short (Fig 3D, top panel, filled diamonds) and WT length (Fig 3D, bottom panel, filled diamonds) telomeres in WT cells. In contrast to Est2 and Est1, levels of Yku80 telomere binding in *pif1-m2* cells were not significantly different from WT binding at either the short (Fig 3D, top panel, open diamonds) or WT length telomeres (Fig 3D, lower panel, open diamonds). Thus, higher telomerase binding to short telomeres is Pif1 dependent. This result is unlikely to be an artifact of telomeres being longer in *pif1-m2* than in WT cells, as the level of binding of telomerase to the VI-R telomere in both the control and experimental strains and to the VII-L telomere in the control strain was similar in WT and *pif1-m2* cells (Fig 3). Likewise, levels of Yku80 binding were not affected by the presence of Pif1 (Fig 3D).

### Pif1 binds preferentially to long telomeres

In WT cells, telomerase preferentially binds [8, 9] and lengthens [6, 7] short telomeres. However, this binding preference was not detectable in *pif1* cells (Figs 3B and S3). Because Pif1 uses its ATPase activity to remove telomerase from DNA ends [22], these results could be explained if Pif1 preferentially removes telomerase from WT length and/or long telomeres. This model predicts that the average length of telomeres that are Pif1-associated will be longer than the average for bulk telomeres.

**Fig 4:**
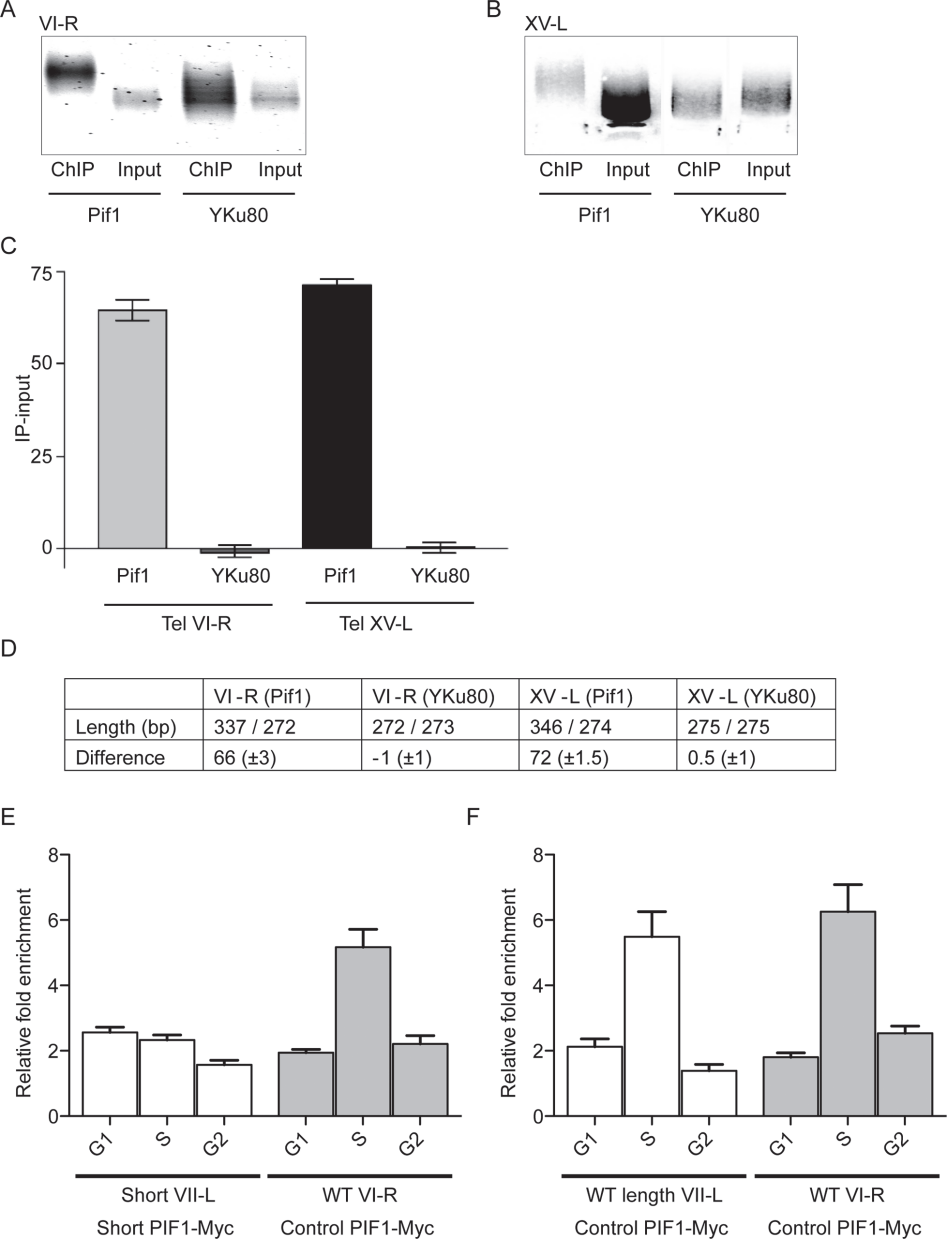
Pif1 preferentially binds to long telomeres. Panels A-D show results from ChIP experiments on two diploid strains that had WT length telomeres but were heterozygous at the *PIF1* locus (*PIF1*/*pif1-K264A*). The only difference between the two strains is that one expressed Pif1-K264A-Myc and the other expressed Yku80-Myc. DNA was either prepared directly from the two strains (Input) or after immuno-precipitation with anti-Myc antibody (ChIP). Both input and ChIP samples were C-tailed and amplified by PCR using primers for telomere VI-R or XV-L and then separated on 1.8% agarose gels. Input and ChIP amplified DNA after gel purification from telomere VI-R (A) or XV-L (B). (C) Graphical presentation of the average difference in bps in lengths of DNA in ChIP and Input samples from the two strains (Pif1 and YKu80) and at both tested telomeres (VI-R and XV-L). Error bars represent one standard deviation from the average for four independent experiments. Using a student’s t-test, the differences between the average telomere lengths of the Input versus ChIP samples were significant for cells expressing Pif1-K264A-Myc (p<0.0001 for both VI-R and XV-L) but not for cells expressing Yku80-Myc (p = 0.9 for VI-R; p = 0.8 for XV-L). (D) The first row shows the average length of telomeres in the two samples (ChIP sample/input sample) for both telomeres in the two strains. The second row shows the average difference in telomere length (length in ChIP minus length in input samples) for the two strains and telomeres. (E and F) Pif1 binding to WT and short telomeres in the inducible VII-L short telomere (panel E) and control WT length VII-L (panel F) strains. Both strains express Pif1-Myc. Both panels show averages ± SD from three experiments. Data are presented as average binding in G1 phase (time points 0, 15 and 30 min; see Fig 3), S phase (time points 45 and 60), and G2 phase (time points 75 and 90). In the experimental strain (panel E), Pif1 binding in S phase was twice as high to the WT VI-R telomere than to the short VII-L in the same cells (p = 0.007; two-tailed unpaired *t*-tests). In the control strain (panel F), Pif1 bound equally well in S phase to WT length VII-L and WT VI-R telomeres (p = 0.5; two-tailed unpaired *t*-tests). In both strains, Pif1 binding to WT length telomeres was significantly higher in S phase than in G1 or G2 (p <0.009; two-tailed unpaired *t*-tests).

To test this possibility, we determined the average size of telomeric DNA in an anti-Pif1 immuno-precipitate (Fig 4). We epitope-tagged a mutant version of Pif1, Pif1-K264A, in which the invariant lysine in the Walker A box is mutated to alanine. Although Pif1-K264A is helicase dead *in vivo* and *in vitro* [18], it binds single-stranded DNA as well as WT Pif1 [22]. We used Pif1-K264A because it gives a stronger signal in ChIP than WT Pif1, probably because it is trapped at its binding sites [29]. However, *pif1-K264A* cells have long telomeres, and the experiment to determine the lengths of Pif1-bound telomeres must be done in cells with WT telomere length. Because the effects of *pif1-K264A* on telomere length are recessive [18], we did the experiment in a heterozygous diploid (*pif1-K264A-Myc*/*PIF1*). As a control, we monitored the lengths of telomeres in an anti-Yku80 immuno-precipitate in a *pif1-K264A*/*PIF1* diploid background.

Chromatin was prepared from both diploid strains and processed for PCR amplification either before (input samples) or after (ChIP samples) immuno-precipitation. After C-tailing the purified DNA, PCR primers were used to amplify either the VI-R (Fig 4A) or XV-L (Fig 4B) telomeres. The PCR-amplified DNA in the input and immuno-precipitated samples was gel separated (Fig 4A and 4B), and the average sizes of the DNA in these samples were determined using an AlphaImager 3400 Molecular Weight Analysis program (Fig 4A, VI-R; B, XV-L).

For both the VI-R and XV-L telomeres, the average size of telomeric DNA in the anti-Pif1 immuno-precipitate was significantly longer in the ChIP samples compared to input DNA (the average size in bps ± SD of telomeric DNA in the ChIP samples was 337.1± 2.0 for VI-R and 346.2 ± 3.4 for XV-L; the average size of input telomeric DNA was 272.7 ± 1.1 for VI-R and 274.0 ± 2.0 for XV-L; Fig 4C and 4D; see Fig 4 legend for p values). As expected for the strain expressing Yku80-Myc [12], the average sizes in bps of telomeric DNA in the ChIP and input samples were indistinguishable (averages ± SD for ChIP samples were 273.3 ± 0.8 and 275.1 ± 1.3, respectively, for telomeres VI-R and XV-L). Thus, Pif1 binds preferentially to long telomeres. This effect is specific for Pif1 because Yku80 binding showed no length preference in the same assay (Fig 4A–4D).

To extend the finding that Pif1 binds preferentially to longer telomeres, we monitored Pif1-Myc binding to short and WT length telomeres using the inducible short telomere system. In both the experimental and control strains, Pif1 telomere binding occurred in S phase. However, in different experiments, the peak of Pif1 binding to a given telomere occurred at different times in S phase. Therefore, for each telomere in both strains, the ChIP binding is presented as average binding from three independent experiments in G1 phase (pooling 0, 15 and 30 min time points), S phase (pooling 45 and 60 min time points), and G2 phase (pooling 75 and 90 min time points) (Fig 4E and 4F). In both, the experimental and control strains, Pif1 binding to all telomeres was low in G1 and G2 phase. In the experimental strain, Pif1 binding was similarly low in S phase to short VII-L. In contrast, binding of Pif1 to the WT length VI-R telomere in both the control and experimental strains and to the WT VII-L telomere in the control strain was three times higher in S phase (Fig 4E and 4F). Thus, using a very different assay, Pif1 binds more robustly to longer telomeres.

## Discussion

In *S*. *cerevisiae* and mammals, short telomeres are preferentially elongated by telomerase [6, 7, 30]. This preference is explained in part by proteins that bind preferentially to short telomeres, such as the MRX complex [12] and Tel1[8, 9, 11]. The presence of these (and other) proteins in combination with low levels of Rif2 result in the preferential binding of telomerase to short telomeres [8, 12, 15]. Here, we show that telomere binding of Pif1, a negative regulator of telomerase, is also length dependent with longer telomeres having higher binding (Fig 4).

*In vivo* and *in vitro*, Pif1 removes telomerase from DNA ends without affecting binding levels of telomere structural proteins [22]. Here we show that Pif1 acts similarly at DSBs (Fig 1) because in the absence of Pif1, Est1 (Fig 1D) and especially Est2 (Fig 1C) bound at higher levels to an induced DSB. In contrast, Cdc13 binding to the break (Fig 1E) was not Pif1-sensitive. Thus, the ability of Pif1 to inhibit telomere addition to spontaneous DSBs [19, 20] can be explained by Pif1 removing telomerase from these breaks.

Pif1-mediated removal of telomerase could affect the frequency of telomerase action, its processivity, or its preference for short telomeres. We used two different methods to determine the impact of Pif1 on these events. STEX experiments indicate that telomerase is more processive *in vivo* in the absence of Pif1, as telomerase added an average of 72 nt (*pif1Δ*) versus 45 nt (WT) to the VII-L telomere in a single cell cycle (Fig 2). Consistent with this finding, Pif1 reduces telomerase processivity *in vitro* [22], and telomeres are longer in *pif1-m2* and *pif1Δ* cells [19].

STEX also revealed that the fraction of telomeres acted upon by telomerase *in vivo* was almost four times higher in *pif1Δ* (28%) versus WT (7.2%) cells (Fig 2). In contrast, *in vitro*, Pif1-dependent release of Est2 from telomeric oligonucleotides increases the fraction of elongated oligonucleotides by freeing Est2 from its original substrate [22] to which it is otherwise tightly bound [31]. The fact that the fraction of telomeres lengthened in the presence of Pif1 is lower *in vivo* and higher *in vitro* is likely explained by the high concentration of telomeric oligonucleotides *in vitro* versus the small number of telomeres in cells. In addition, as Est1 and Est3 binding to telomeres is limited to a short period late in S phase [4, 5], telomerase action occurs only during a narrow window of the cell cycle [2, 3] while the standard *in vitro* system is Est1 and Est3-independent [32].

To determine if Pif1 affects the preferential lengthening of short telomeres, we used an assay in which a single short telomere is induced in cells with otherwise WT length telomeres [6]. This assay was used previously to show that in WT cells, Est2 and Est1 bind preferentially to short telomeres [8, 9]. However, in *pif1-m2* cells, we see similar levels of Est2 and Est1 at short and WT length telomeres (Figs 3B and S3). This loss of preferential binding of Est2 and Est1 to the short VII-L telomere in *pif1-m2* cells is unlikely to be a consequence of all telomeres being longer in *pif1-m2* cells [18, 19]. Even though the average length of the “short” VII-L telomere (±SD) (196 ± 11.7 bp) was longer in *pif1-m2* versus WT (124.8 ± 7.2 bps) cells (S2B Fig), 196 bp is still short enough to be preferentially lengthened by telomerase in WT cells [7]. Moreover, levels of Est2 binding to the control VI-R telomere were not affected by Pif1 (nor was Yku telomere binding) (Fig 3C and 3D). Thus, our results cannot be explained by its being more difficult to ChIP Est2 to longer telomeres. Finally, in the control strains the difference in the average post-recombination lengths of the VII-L telomeres in WT versus *pif1-m2* cells was even larger (71 bp difference in experimental versus 78 bp in control strains) (S2B Fig), yet the levels of Est2 association in the control strains were not *PIF1*-sensitive (Fig 3C and 3D). Together with the STEX results, our data demonstrate that Pif1 contributes to the preferential targeting of telomerase to short telomeres.

If Pif1 binds more readily to long telomeres, it could explain how Pif1 contributes to preferential telomerase activity at short telomeres. To test this possibility, we determined the average length of Pif1-associated telomeres. For both, the VI-R and XV-L telomeres, Pif1-associated telomeres were about 70 bps longer than bulk telomeres (Fig 4D). In contrast, using the same assay, Yku80-associated telomeres were the same length as bulk telomeres in the two strains. In addition, in S phase, Pif1 bound better to WT length telomeres than to a very short telomere (Fig 4E and 4F). These data argue that Pif1 contributes to the selective lengthening of short telomeres by binding to and removing telomerase preferentially from longer telomeres. This finding is interesting in light of an *in vitro* study that used single molecule analyses to show that Pif1 was better able to displace telomerase from substrates with long TG_1-3_ single-strand tails [33]. If longer telomeres have longer G-tails *in vivo*, the two observations may be linked. Rif2, another negative regulator of yeast telomerase, also binds to a greater extent at wild type than to short telomeres [12]. Thus, preferential lengthening of short telomeres is achieved by proteins like Pif1 and Rif2 that act preferentially at longer telomeres and proteins like MRX, Tel1, and Tbf1 that act preferentially at shorter telomeres. It is tempting to speculate that Rif2 recruits Pif1 preferentially to long telomeres.

Pif1 efficiently binds to and unwinds G4 structures *in vitro* and suppresses DNA damage at G4 motifs *in vivo* (reviewed in [16]; also, [23–25]). Thus, a unifying model for Pif1 action is that it inhibits telomerase by unwinding telomeric G4 structures. However, intra-molecular G4 structures inhibit telomerase [34]. Thus, it is difficult to attribute the inhibitory effects of Pif1 on telomerase to its G4-unwinding activity. However, the presence of G4 structures might stimulate Pif1’s ability to displace telomerase as it does Pif1 unwinding of duplex DNA [35].

Another possibility is that Pif1 uses its ATPase activity to displace the protein subunits of telomerase from DNA ends, the type of protein eviction activity attributed to the closely related *S*. *cerevisiae* Rrm3 and the *S*. *pombe* Pfh1 helicases during chromosome replication [36, 37]. Although we can not rule out this model, it seems unlikely as Pif1 does not affect Cdc13 [22] or Yku (Fig 3D) telomere binding. Rather we favor a model where Pif1 removes telomerase from telomeres by disrupting the telomerase RNA-telomeric DNA intermediate as Pif1 unwinds RNA/DNA hybrids very efficiently [38, 39].

In summary, the Pif1 helicase affects multiple aspects of the telomerase reaction: it reduces telomerase processivity, the frequency of telomere elongation, and the preference of telomerase for short telomeres. All of these effects could be a result of Pif1 binding preferentially to (and hence telomerase removal from) telomeres that are longer than average in length. Because yeast telomerase is not abundant, with fewer telomerase complexes than telomeres [5, 40, 41], this regulation is important to ensure that those telomeres most in need of lengthening receive telomerase.

## Materials and Methods

### Yeast strains and growth conditions

Strains and primers are presented in, respectively, Tables 1 and 2. Experiments were carried out in *RAD5* ^*+*^ versions of W303, unless otherwise indicated. Deletions eliminated entire ORFs. The *pif1-m2* and *pif1-K264A* strains were made as in [19]. Epitope tagging to generate Est1-G8-Myc9, Est2-G8-Myc18, Cdc13-Myc9, Yku80-G8-Myc18, Pif1-Myc13 and Pif1-K264A-Myc13 was carried out as described [8, 29, 42, 43]. Each Myc-tagged protein was expressed from its endogenous locus and promoter. Except for the experiments using Myc-Pif1-K264A, the tagged protein was the only form of the protein in cells. STEX strains contained *URA3* adjacent to the VII-L telomere [44]. The strains used to analyze telomerase binding as a function of telomere length contained a galactose-inducible *FLP1* and a modified chromosome VII-L for excision of telomere-adjacent DNA [6].

**Table 1:** Strains used in this study.

Strain	Genotype	Source
JP192	W303 MATa/Δ *RAD5/RAD5 tlc1*Δ::*HIS3*/+ *VII-L*::*URA3*/+	This study
JP223	W303 MATa/Δ *RAD5/RAD5 tlc1Δ*::*HIS3*/+ *pif1 Δ*::*TRP1*/+ *VII-L*::*URA3*/+	This study
LEV187	W303 MATa *cir* ^*0*^ *leu2*::*LEU2-Gal-FLP adh4*::*FRT-URA3-FRT*	[6]
LEV220	W303 MATa *cir* ^*0*^ *leu2*::*LEU2-Gal-FLP adh4*::*FRT-URA3-(TG*_*270*_ *)*_*2*_ *-FRT*	[6]
JP350	LEV220 *RAD5 EST2-G8-Myc18*::*TRP1 bar1Δ*::*kan*	This study
JP356	LEV220 *RAD5 EST2-G8-Myc18*::*TRP1 bar1Δ*::*kan pif1-m2*	This study
JP382	LEV187 *RAD5 EST2-G8-Myc18*::*TRP1 bar1Δ*::*kan*	This study
JP380	LEV187 *RAD5 EST2-G8-Myc18*::*TRP1 bar1Δ*::*kan pif1-m2*	This study
JP424	LEV220 *RAD5 KU80-G8-Myc18*::*TRP1 bar1Δ*::*kan*	This study
JP426	LEV220 *RAD5 KU80-G8-Myc18*::*TRP1 bar1Δ*::*kan pif1-m2*	This study
YAB285	W303A *matΔ RAD5 ade2Δ lys2Δ leu2*::*Gal-HO mnt2*::*LYS2 adh4*::*ADE2-TG80-HOsite*	[26]
JP281	YAB285 *EST2-G8-Myc18*::*TRP1 bar1Δ*::*nat*	This study
JP325	YAB285 *EST2-G8-Myc18*::*TRP1 bar1Δ*::*nat pif1-m2*	This study
JP334	YAB285 *EST1-G8-Myc9*::*TRP1 bar1Δ*::*nat*	This study
JP337	YAB285 *EST1-G8-Myc9*::*TRP1 bar1Δ*::*nat pif1-m2*	This study
JP370	YAB285 *CDC13-Myc9*::*TRP1 bar1Δ*::*nat*	This study
JP372	YAB285 *CDC13-Myc9*::*TRP1 bar1Δ*::*nat pif1-m2*	This study
KP222	W303 MATa/Δ *RAD5/RAD5 pif1K264A-Myc13*::*KanMx*/+ *TRP1 bar1Δ*::*TRP1/+*	This study
KP238	W303 MATa/Δ *RAD5/RAD5 KU80-G8-Myc18*::*TRP1/+ pif1K264A*/+	This study
AC262	LEV220 diploid, *Pif1-Myc13-TRP/Pif1-Myc13-TRP RAD5/rad5-535 bar1D*::*Kan/bar1*::*Kan*	This study
AC261	LEV187, Pif1-Myc13::TRP, rad5/535, *bar1Δ*::*TRP1*	This study

**Table 2:** Primers used in this study.

Name	Sequence
TEL7L	GACATTATTATTGTTGGAAGAGGACTATTTGC
PolyG18	CGGGATCCGGGGGGGGGGGGGGGGGG
FTM7*	TGGTTTCTTGTCTGGTTTCTCAAC
RTM7*	GAATACGCTGGTTTGCATAAAGG
RT-ARO+	TCGTTACAAGGTGATG
RT-ARO-	AATAGCGGCAACAAC
VI-R+	ATCATTGAGGATCTATAATC
VI-R-	CTTCACTCCATTGCG
FRT+**	TGATATGTGTTACGCAGAATAC
FRT-**	TGAGAAGCACCGCAATG
pif1D-F	CTGGTATTGCCTCGATATTTTTATTTGTAATATTATCCATTGAGCGATTAGCTTACTTGTATCAATCAATTTTACCGTTTCGGTGATGAC
pif1D-R	CCGCGGAACTATGTATCTCGGCCTTGATTATTATAGCAGTTTGTATTCTATATAACTATGTGTATTAATATGTACTTCCTGATGCGGTATTTTCTCCT
bar1D-F	ATTTAATTCTAGTGGTTCGTATCGCCTAAAATCATACCAAAATAAAAAGAGTGTCTAGAAGGGTCATATACGTACGCTGCAGGTCGACGGA
bar1D-R	GCTTTCCATGTATTAAAAATGACTATATATTTGATATTTATATGCTATAAAGAAATTGTACTCCAGATTTCCATCGATGAATTCGAGCTCGT

* [26];

**[8]

Strains for DSB experiments had a galactose-inducible *HO* gene and a modified chromosome VII-L with the HO endonuclease recognition site between *ADH4* and *MNT2* [26]. A heterozygous (*pif1-K264A/PIF1*) diploid strain in which either Pif1-K264A or Yku80 was epitope tagged was used to determine the lengths of Pif1-associated telomeres. For experiments using galactose, cells were grown in 3% raffinose prior to induction in 1% galactose. For HO experiments, cells were maintained on media lacking lysine to prevent leaky expression from the *HO* gene.

### Single telomere extension (STEX) assays

Recipient cells were prepared from freshly dissected spore clones from JP192 (*tlc1Δ*::*HIS3 PIF1*) or JP223 (*tlc1Δ*::*HIS3 pif1Δ*::*TRP1*) and grown to OD_660_ 0.2. Next, 6 x 10^7^ recipient cells were mated with 1 x 10^7^ of either WT or *pif1Δ*::*TRP1* donor cells freshly dissected from JP192 or JP223. Mating mixtures were filtered onto four membranes (Microfil S, Millipore), placed onto pre-warmed YEPD plates, and incubated at 30°C for 3 h [7]. Cells were resuspended in 30 ml synthetic media lacking histidine (YC-his) and then added to 90 ml of fresh YC-his. Fifty ml resuspended mating cells were taken immediately for analysis; the remaining cells were incubated at 30°C with shaking at 160 rpm. After 2 h (5 h after initial mixing), an additional 50 ml were taken for analysis. Mating efficiency was monitored by flow cytometry by determining the proportion of cells containing >2N DNA content after correcting for the ratio of recipient to donor cells in the mating mixture (i.e., 100% mating of recipient cells results in 16.7% of total cells with >2N DNA content). Reported experiments had >85% mating efficiency. DNA was isolated using Masterpure Yeast DNA Purification Kits (Epicentre). Telomere PCR was performed using 400 ng of DNA as described [7], except that primers TEL7L and PolyG18, specific to the *URA3*-marked telomere VII-L, were used for amplification (Table 2). PCR products were ligated into the pDRIVE vector (Qiagen) as per the manufacturer’s instructions. Plasmid inserts were sequenced (Genewiz, South Plainfield, NJ) using the SP6 primer. Sequences were aligned by eye and analyzed using the contig assembler of Vector NTI (Invitrogen). Where indicated, Fisher’s Exact tests (which test potential relationships between categorical variables) and Mann-Whitney U tests (non-parametric tests of significance for two independent sample groups) were used to determine statistical significance.

### Cell synchrony and induction of telomere shortening or cutting at HO site

Strains used for analysis of protein association at either a short telomere or a double strand break were grown overnight at 30°C in rich media plus 3% raffinose to OD_660_ 0.15. Synthetic Δ-factor was added (final concentration 160 μM), and cells were incubated at 30°C for ~2 h or until > 90% of cells were unbudded (G1 phase). Samples were then taken for flow cytometry, Southern blot analysis, and ChIP (“0-gal” time point). Dry galactose (Sigma) was added to the rest of the culture (final concentration 1%) to induce either the *FLP* recombinase or the *HO* endonuclease; cells were incubated at 30°C for 3 h [8]. Cells were filtered and then resuspended in YEPD with a final concentration of 160 μM Δ-factor and incubated for 15 min at 24°C. The cells were filtered again and resuspended in rich medium containing glucose (no Δ-factor) and 170 μg/ml P6911 Protease (Sigma) and released into the cell cycle at 24°C. Samples were taken every 15 min for one cell cycle and analyzed by flow cytometry, Southern blot, and ChIP [45].

### Chromatin immunoprecipitation (ChIP)

ChIP was performed as described [46], except that cells were lysed by adding 10 μl 5 mg/ml Zymolyase 100T (MP Biomedicals) to thawed samples, which were then incubated at 37°C for ~10 min or until visual inspection showed >90% lysis. Myc-tagged proteins were immunoprecipitated using an Δ-Myc monoclonal antibody (Clontech). Immunoprecipitated DNA was quantitated by real-time PCR on an iCycler iQ Real-Time PCR Detection System (Bio-Rad Laboratories) as in [45], except that primers were used to amplify either a region adjacent to a short telomere on chromosome VII-L (primers FRT+ and FRT- [8]) and WT telomere VI-R (primers VI-R+ and VI-R-) or a region adjacent to an *HO* recognition site (primers FTM7 and RTM7 [26]). ChIP samples were normalized to inputs; data are presented as fold enrichment relative to a non-telomeric control site on chromosome IV (*ARO1*, using primers RT-ARO+ and RT-ARO). Each synchrony was repeated a minimum of three times to obtain an average enrichment value. Error bars represent the standard deviation from three or more independent experiments. A two-tailed students t-test was used to determine statistical significance (p≤0.05).

### Determining lengths of Pif1-associated telomeres

After ChIP from asynchronously growing diploid strains, telomere PCR was performed with minor modification of the methods in [12]. Briefly, ChIP samples were C-tailed according to manufactures instructions using T4 polynucleotide kinase (Invitrogen). PCR conditions were 58°C annealing for 30 s and 72°C extension for 45 s. The PCR products were separated on a 1.8% (w/v) MetaPhor (Lonza) agarose gel. A subset of the DNA was cloned and sequenced to establish that it was indeed telomeric DNA: of the 90 sequenced clones, 89% were telomeric DNA while the remainder had little or no insert DNA. The AlphaImager 3400 Molecular Weight Analysis program was used to determine the average telomere length in each sample. Experiments were performed in three biological replicates; telomere length was determined at two different telomeres (VI-R and XV-L).

## Supporting Information

S1 Fig The *HO* endonuclease cleaves efficiently at TG80-adjacent HO recognition sites. Cleavage at the HO recognition site was monitored by Southern blotting, similar to [8]. Concurrent with all ChIP experiments, samples of cells were taken before and after galactose induction of HO endonuclease for DNA purification and Southern blot analysis. *Eco*RV-digested DNA was resolved on 1% agarose gels, transferred to a nitrocellulose membrane (Amersham Biosciences), and probed simultaneously with DNA located on the centromere-proximal side of the *HO* site and control DNA from the *NMD5* gene. Representative blots of WT and *pif1-m2* mutant strains are shown. Cutting efficiencies ranged from 65–80%. MW markers are indicated in Kb.
(TIF)

S2 Fig Efficiency of FLP1 recombination and telomere length. Flp1 recombination efficiency and post-recombination telomere length at chromosome VII-L were monitored by Southern blotting, as in [8]. Representative Southern blots are shown. **(A)** Recombination efficiency was determined. DNA samples taken from inducible short and recombination control VII-L strains (wild type [WT] and *pif1-m2* [*-m2*] cells) before and after galactose induction were digested with *Eco*RV and *Xho*I, resolved on 1% agarose gels, and transferred to a nitrocellulose membrane (Amersham Biosciences). Blots were probed with a radiolabeled *ADH4* fragment. Using ImageQuant, the amount of radioactivity in each band was quantitated. Recombination efficiency was calculated by analyzing the unrecombined band. Recombination efficiencies were at least 75% in each strain. **(B)** VII-L telomere lengths after Flp1 action were determined using samples taken after galactose addition. DNA was digested with *Stu*I, which liberates the VII-L telomere as a smear of telomeric DNA plus 929 bp of internal sequence. ImageQuant was used to determine the size of each telomere. In the experimental strains, the average ± SD lengths of the “short” VII-L telomere in WT and *pif1-m2* cells was, respectively, 124.8 ± 7.2 and 196 ± 11.7 bp. In the control strains, the average (range) post-recombination lengths of the VII-L telomeres in WT and *pif1-m2* cells were, respectively, 328 (23.7) and 406 (5.0) bp (for the control strain, telomere lengths were determined in two experiments; corresponding ChIP results in Fig 3 were obtained from three independent experiments with each strain). MW markers are indicated in Kb.
(TIF)

S3 Fig Level of Est1 binding to short telomeres is Pif1 dependent. **(A)** Schematic of strain used to generate short telomere. Methods are identical to those in Fig 3 of main text except that Est1 telomere binding was analyzed. The identities of the telomeres being studied are indicated in the upper left corner of each graph. Est1-Myc binding to the short VII-L telomere **(B)** or the WT length VI-R telomere **(C)** in *PIF1* (closed circles) or *pif1-m2* (open circles) cells. The values for Est1-Myc binding in *pif1-m2* cells are an average of three independent experiments; error bars are one standard deviation. Binding of Est1-Myc in the WT strain was done once, but the values through the cell cycle were identical to published data [8], which we used for statistical comparison. Binding of Est1-Myc at short telomere VII-L was 2.8 fold lower in *pif1-m2* compared to WT during S phase (45min, p = 0.01; 60 min, p = 0.001; two-tailed unpaired *t*-tests). In contrast binding to the WT length VI-R telomere was similar in *pif1-m2* and WT cells during S phase (p >0.05; two-tailed unpaired *t*-tests).
(TIF)
