# Supplementary figures and images for: The Pif1 Helicase, a Negative Regulator of Telomerase, Acts Preferentially at Long Telomeres
Source: PLoS Genet. 2015 Apr 23;11(4):e1005186. doi: 10.1371/journal.pgen.1005186 (PMC4408051; doi:10.1371/journal.pgen.1005186)

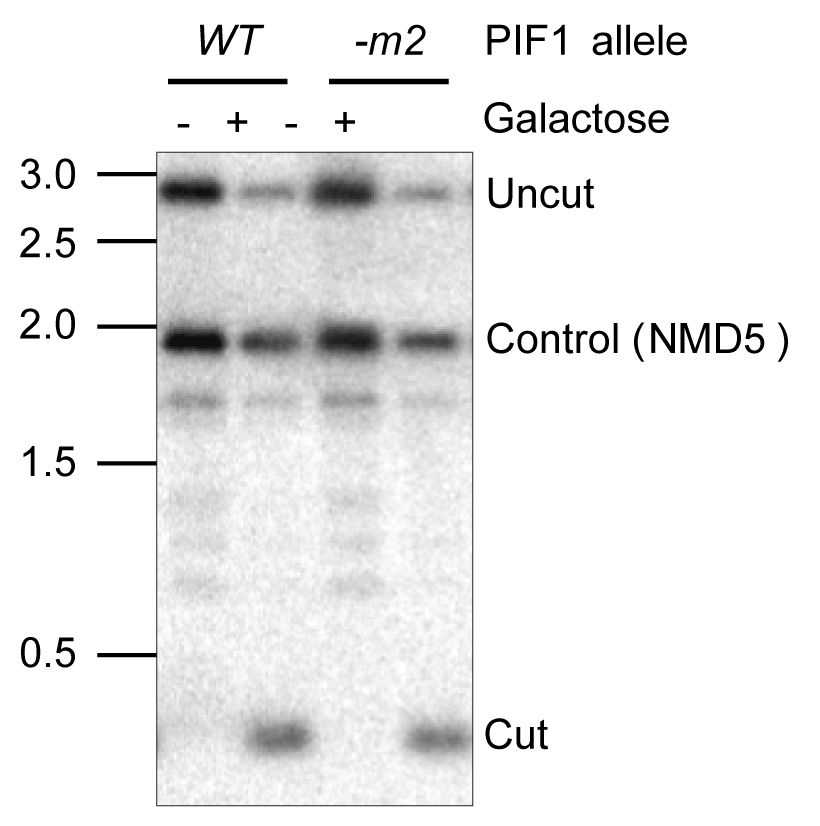

Supplement: S1 Fig — Cleavage at the HO recognition site was monitored by Southern blotting, similar to [8]. Concurrent with all ChIP experiments, samples of cells were taken before and after galactose induction of HO endonuclease for DNA purification and Southern blot analysis. EcoRV-digested DNA was resolved on 1% agarose gels, transferred to a nitrocellulose membrane (Amersham Biosciences), and probed simultaneously with DNA located on the centromere-proximal side of the HO site and control DNA from the NMD5 gene. Representative blots of WT and pif1-m2 mutant strains are shown. Cutting efficiencies ranged from 65–80%. MW markers are indicated in Kb. (TIF) [file pgen.1005186.s001.tif]

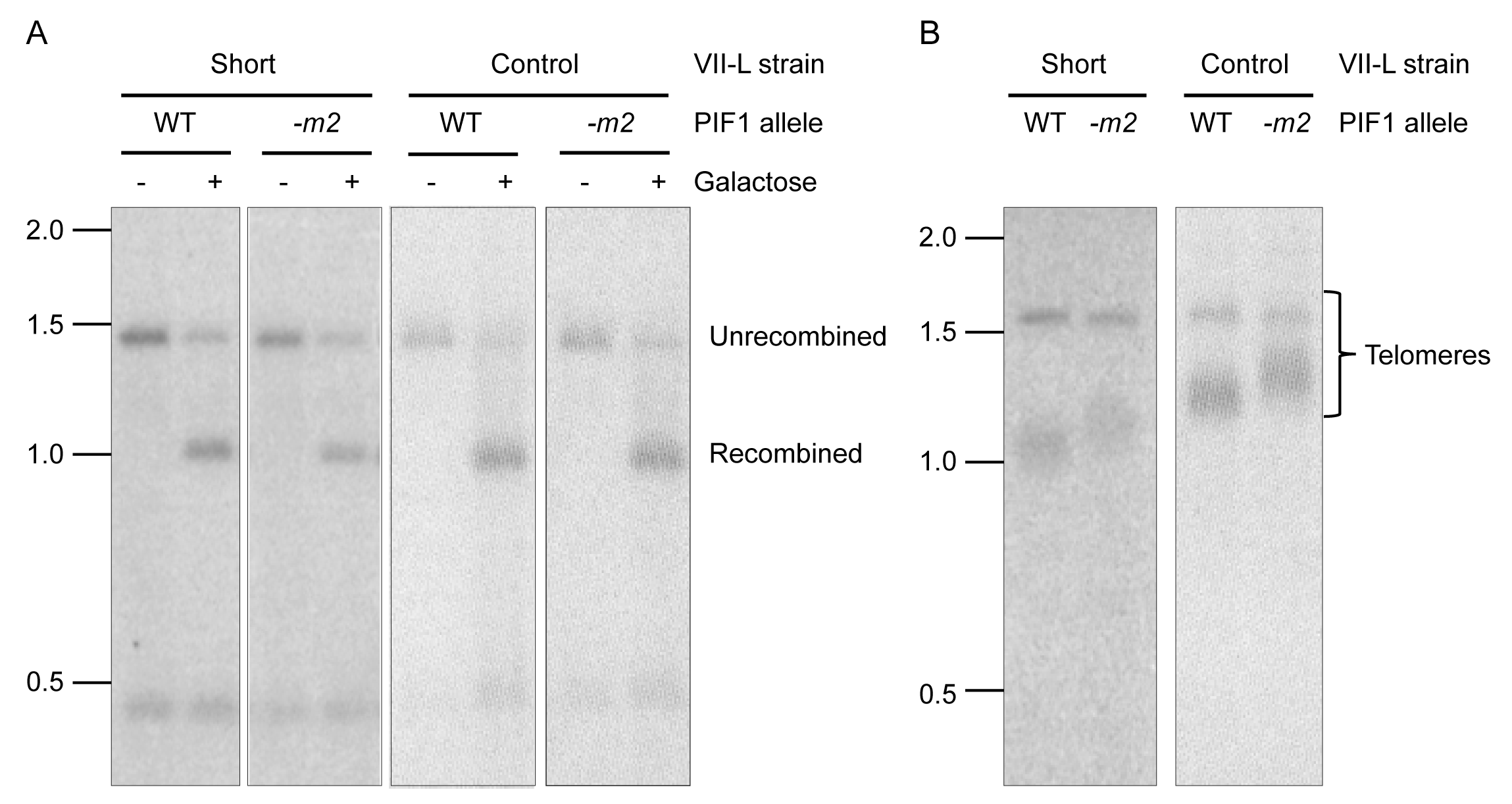

Supplement: S2 Fig — Flp1 recombination efficiency and post-recombination telomere length at chromosome VII-L were monitored by Southern blotting, as in [8]. Representative Southern blots are shown. (A) Recombination efficiency was determined. DNA samples taken from inducible short and recombination control VII-L strains (wild type [WT] and pif1-m2 [-m2] cells) before and after galactose induction were digested with EcoRV and XhoI, resolved on 1% agarose gels, and transferred to a nitrocellulose membrane (Amersham Biosciences). Blots were probed with a radiolabeled ADH4 fragment. Using ImageQuant, the amount of radioactivity in each band was quantitated. Recombination efficiency was calculated by analyzing the unrecombined band. Recombination efficiencies were at least 75% in each strain. (B) VII-L telomere lengths after Flp1 action were determined using samples taken after galactose addition. DNA was digested with StuI, which liberates the VII-L telomere as a smear of telomeric DNA plus 929 bp of internal sequence. ImageQuant was used to determine the size of each telomere. In the experimental strains, the average ± SD lengths of the “short” VII-L telomere in WT and pif1-m2 cells was, respectively, 124.8 ± 7.2 and 196 ± 11.7 bp. In the control strains, the average (range) post-recombination lengths of the VII-L telomeres in WT and pif1-m2 cells were, respectively, 328 (23.7) and 406 (5.0) bp (for the control strain, telomere lengths were determined in two experiments; corresponding ChIP results in Fig 3 were obtained from three independent experiments with each strain). MW markers are indicated in Kb. (TIF) [file pgen.1005186.s002.tif]

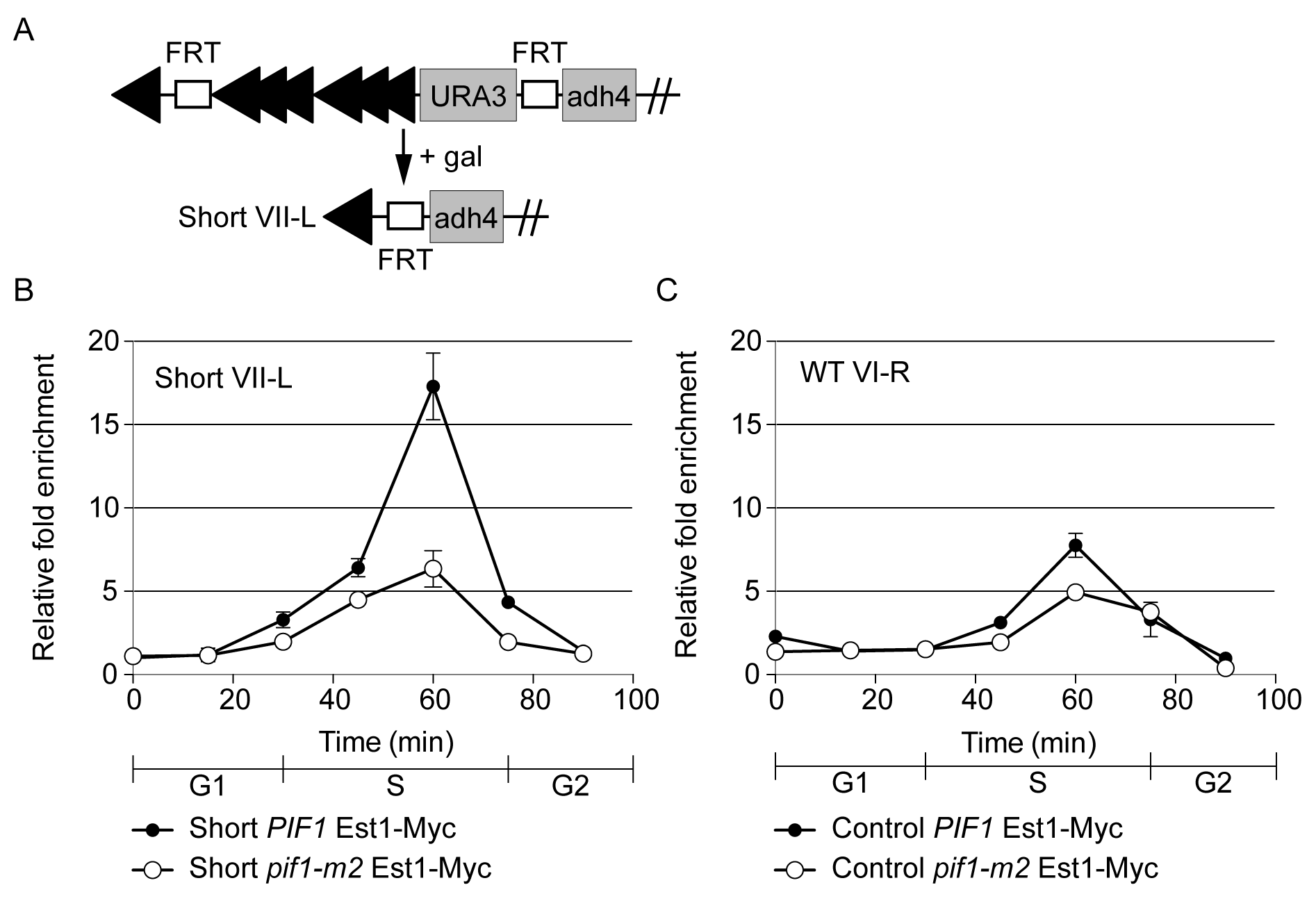

Supplement: S3 Fig — (A) Schematic of strain used to generate short telomere. Methods are identical to those in Fig 3 of main text except that Est1 telomere binding was analyzed. The identities of the telomeres being studied are indicated in the upper left corner of each graph. Est1-Myc binding to the short VII-L telomere (B) or the WT length VI-R telomere (C) in PIF1 (closed circles) or pif1-m2 (open circles) cells. The values for Est1-Myc binding in pif1-m2 cells are an average of three independent experiments; error bars are one standard deviation. Binding of Est1-Myc in the WT strain was done once, but the values through the cell cycle were identical to published data [8], which we used for statistical comparison. Binding of Est1-Myc at short telomere VII-L was 2.8 fold lower in pif1-m2 compared to WT during S phase (45min, p = 0.01; 60 min, p = 0.001; two-tailed unpaired t-tests). In contrast binding to the WT length VI-R telomere was similar in pif1-m2 and WT cells during S phase (p >0.05; two-tailed unpaired t-tests). (TIF) [file pgen.1005186.s003.tif]
